# An Implementer's Perspective on Vouchers for Sexual and Reproductive Health Services

**DOI:** 10.9745/GHSP-D-16-00373

**Published:** 2016-12-23

**Authors:** Matthew Wilson, Caitlin Mazzilli

**Affiliations:** a Marie Stopes International, London, UK.

See related article by Menotti and Farrell.

We found the commentary on vouchers by Menotti and Farrell, published in the September issue of GHSP,^1^ thought-provoking and comprehensive. At Marie Stopes International (MSI), every year we deliver half a million voluntary contraception and maternal health services via voucher programs in 8 countries. We have directly experienced that “vouchers can be a highly effective tool to increase access to and use of family planning and reproductive health services, especially for special populations including the poor, youth, and postpartum women.”^1^ We are writing to share an implementer's perspective and a few points of differentiation from Menotti and Farrell.

## PREPARING FOR THE COMPLEXITY CRUCIAL TO SUCCESS

The authors touch upon the complexity of voucher programs, but we believe this point needs to be underscored. Voucher programs are simple to describe, intuitively logical and compelling, but they are challenging to design and implement well and at scale. Despite the growing body of evidence on voucher programs by organizations such as Population Council, many aspects of implementation still need to be better understood. A powerful voucher program requires the perfect sequencing of activities from the supply side and demand side to financial management and controls, but this sequencing can be a challenge for field teams to master. Furthermore, there is appetite for ever-more rigorous evidence to coordinate alongside programming, including evidence that these programs can influence national health financing policy. All can be done, but not everywhere. Without strong capacity, many programs will struggle to cope.

Other implementation challenges that are less appreciated by the literature include how to compensate voucher distributors and set reimbursement rates that genuinely promote choice of contraceptive method; how to harness mHealth opportunities when working with populations that have limited access to mobile technologies or where phone communication about reproductive health may pose an ethical risk; how to conduct verification with youth who are reluctant to give consent to be followed up; and how to influence national health financing policy in conducive market contexts. Importantly, we also need to manage expectations. To manage the complexity, achieve economies of scale, realize a return on the systems investment, and deliver value for money, voucher programs need to be resourced well. And to master the complexity, we need to be tolerant of mistakes and alert and responsive to the learning they provide.

## REMOVING THE FINANCIAL BARRIERS IS JUST ONE WAY IN WHICH VOUCHERS CHANGE BEHAVIOR

Menotti and Farrell pitch voucher programs as a means of removing financial barriers, and indeed they are. That cost is a barrier for many special populations is indisputable. A recent article in the Bulletin of the World Health Organization,^2^ for example, outlined a series of financial barriers to health care, including the influence of financing policies on choice and access to sexual and reproductive health services; the negative impact of direct payments on adolescents' use of health services within health markets at all stages of development; and the influence of direct payments on the type of services used by adolescents.

But just as cost is rarely the only barrier, vouchers increase use of services among special populations not merely because they remove this financial barrier. Our programs have found the “counseling moment” (a phrase we attribute to Anna Gorter) provided by the voucher distributor and the physical possession of a voucher builds clients' self-efficacy and sense of entitlement. This illustrates how important it is that those of us implementing voucher programs consider these financing tools within a broader behavior change framework. Agencies working to enroll populations into expanding universal health coverage schemes may also learn from the approaches that voucher programs have used.

## DEBUNKING THE PERCEPTION THAT VOUCHERS ARE MORE PRONE TO FRAUD

Menotti and Farrell acknowledge the challenge of fraud prevention and detection within voucher programs. While fraud invariably comes up in discussions about vouchers, we caution against assumptions that output-based financing interventions such as voucher programs are more susceptible to fraud than traditional input-based ones. Some studies have found that output- or results-based interventions may actually be less prone to fraud than input-based interventions,^3^ because they are accountable for and oriented toward what can be evidenced and measured. However, a fabricated voucher claim form seems to unsettle us more than a salaried health worker that does not show up for work, leakage of donated and procured commodities, or bid rigging, yet all are fraud and must be controlled as such. Indeed our experience at MSI demonstrates that voucher programs with robust fraud prevention and detection systems can limit fraudulent activity. The strongest voucher programs track every voucher throughout its lifecycle, collect documentation for every service, identify outliers and unusual patterns, and trace clients to verify their existence and eligibility. Inevitably, in many of the contexts where we work, there will always be a residual risk but with proper systems in place this risk should not detract from the potential health impact of voucher programs.

## REORIENTATING VOUCHER PROGRAMS TO CATALYZE HEALTH FINANCING CHANGES

Menotti and Farrell note that “voucher programs may strengthen capacity and readiness in the health system for implementing universal health coverage.”^1^ We concur. This readiness can only come from dialogue that spans far wider than the implementers of the voucher program and its direct donors. The urgency of such engagement is of course dependent on the context, and in most fragile contexts voucher programs will remain a critical means of increasing access to priority services, full stop. In transitioning contexts, we need to continue to test the hypothesis that vouchers can demonstrate a key path toward strategic purchasing. This link might be more obvious to the voucher-acquainted than to the voucher novice. In the political sphere, where many health financing decisions are taken, few voucher program implementers tend to tread. However, unlike complicated reforms, voucher programs are intuitive and might just have political appeal. We need to rethink how to ensure the lessons of bringing coverage to key populations, with quality-assured services and careful payment mechanisms, are not lost as countries grapple with these very questions on the road toward universal health coverage.
